# Beyond Acute EGFR Blockade: Biological Basis and Clinical Evidence for Long-Term Nimotuzumab Therapy

**DOI:** 10.3390/biomedicines14071570

**Published:** 2026-07-14

**Authors:** Tania Crombet Ramos, Arlhee Díaz Miqueli, Rolando Pérez Rodríguez

**Affiliations:** 1 Clinical Research Department, Center of Molecular Immunology (CIM), Havana 16040, Cuba; 2 Oncoscience GmbH, 22869 Schenefeld, Germany; a.diazmiqueli@oncoscience.de; 3 Research Department, Center of Molecular Immunology (CIM), Havana 16040, Cuba; rolando@cim.sld.cu

**Keywords:** nimotuzumab, long-term treatment, density-selective targeting, maintenance, immunomodulation

## Abstract

Nimotuzumab is a humanized anti-EGFR monoclonal antibody with a unique pharmacodynamic profile characterized by intermediate affinity and bivalent binding dependence, enabling density-selective tumor targeting while sparing normal tissues from the severe skin rash and other toxicities common to EGFR inhibitors. Since its first approval in 2002, nimotuzumab has been registered for eight cancer indications. Unlike conventional fixed-dose schedules, emerging evidence supports prolonged administration beyond initial combination therapy. This review summarizes clinical data from pancreatic cancer, esophageal cancer, high-grade glioma, pediatric diffuse intrinsic pontine glioma, head and neck squamous cell carcinoma, nasopharyngeal cancer and other solid tumors, showing that extended nimotuzumab exposure, often as maintenance monotherapy, may prolong overall survival, progression-free survival, and disease control compared to limited cycles. Despite heterogeneity in tumor types and treatment regimens, maintenance nimotuzumab was consistently associated with better results, particularly in terms of overall survival. Notably, significant survival benefits were observed in locally advanced SCCHN (24.9 vs. 12.5 months) and esophageal cancer (15.9 vs. 8.1 months) across independent clinical trials. Mechanistically, nimotuzumab exerts direct cytostatic effects via G1 arrest, potent anti-angiogenic activity through VEGF downregulation, indirect pro-apoptotic effects, and broad immunomodulation including ADCC, NK-DC cross-talk, EGFR-specific CD8+ T cell priming, upregulation of HLA class I, and favorable regulation of regulatory T cells. Its density-selective binding reduces selective pressure for acquired resistance. Future research priorities should include prospective randomized trials specifically evaluating maintenance strategies, biomarker-driven patient selection, the molecular characterization of resistance mechanisms, integration with immunotherapy and modern combination regimens, and the development of next-generation platforms, including antibody–drug conjugates and multi-specific constructs.

## 1. Introduction

Nimotuzumab is a humanized anti-EGFR antibody generated by humanizing the murine monoclonal antibody (mAb) ior-egf/r3 [1,2]. The parental murine antibody was obtained by immunizing Balb/c mice with a purified EGFR fraction from a human placenta and by fusing the mouse splenocytes with a non-secreting hybridoma [1]. This murine antibody was used for clinical research. To reduce the immunogenicity and improve the immune effector function of the antibody, nimotuzumab was humanized by inserting the complementarity-determining regions (CDR) of the murine parental antibody into a human framework [2]. Besides the murine CDRs, other amino acids were back-mutated into the variable fraction of the humanized backbone to grant the desired target affinity [2]. The IgG1 subclass was selected for humanizing the antibody in order to increase its immunological effector function, i.e., antibody-dependent cell cytotoxicity (ADCC) and complement-dependent cytotoxicity (CDC) [2].

The first phase I clinical trial of the humanized antibody started in 1999 [3]. Twelve patients with epithelial tumors received a single dose of nimotuzumab in a study intended to characterize the safety, pharmacological properties and immunogenicity of the antibody [3]. The dose was scaled up from 50 to 400 mg. The antibody was very safe, and unlike the other drugs in its class (antibodies or EGFR tyrosine kinase inhibitors, TKIs), nimotuzumab did not induce skin rash, folliculitis, hepatotoxicity, or hypomagnesemia. In serial biodistribution images in patients, nimotuzumab exhibited high tumor uptake and retention, along with rapid clearance from normal tissues [3].

## 2. Approved Registrations

Nimotuzumab obtained its first registration in 2002. Since then, it has been approved in more than 20 countries worldwide for several indications. Nimotuzumab approval indications in Cuba (the country of origin) are summarized in Table 1.

For oncogene-addicted tumors, the optimal duration of targeted therapy is not universally defined; in advanced disease, treatment is generally continued until progression or intolerance, whereas in early-stage disease, the current evidence supports fixed adjuvant courses. This narrative review shows the clinical evidence supporting the potential benefits of long-term treatment with nimotuzumab and the biological rationale underlying this therapeutic approach.

## 3. Which Clinical Evidence Supports the Long-Term Use of Nimotuzumab?

Although no randomized trial has directly compared short vs. long nimotuzumab courses, the available evidence suggests that prolonged administration in patients with locally advanced squamous cell carcinoma of the head and neck (SCCHN), nasopharyngeal carcinoma (NPC), esophageal cancer, brain tumors, and pancreatic adenocarcinoma is associated with durable disease control, extended progression-free survival (PFS) and overall survival. However, in the absence of prospective comparisons between limited and prolonged treatment schedules, these observations should be interpreted with caution and considered hypothesis-generating.

In this manuscript, we postulate that the limited-cycle treatment is not the optimal approach for cancer control. This assumption is reinforced by a plausible scientific rationale. The following sections summarize the available evidence for prolonged nimotuzumab use across several tumor types and clinical trials. In particular, in head and neck and esophageal cancer scenarios, separate trials evaluated either six doses or maintenance schedules in similar patient populations and treatment regimens; in both settings, continuous nimotuzumab was well tolerated and associated with better clinical outcomes.

## 4. Squamous Cell Carcinoma of the Head and Neck and Nasopharyngeal Cancer

The first randomized study of nimotuzumab in locally advanced squamous cell carcinoma of the head and neck (SCCHN) evaluated the addition of only six doses of nimotuzumab to the conventional radiotherapy. In this double-blind, placebo-controlled trial of 106 patients with unresectable stage III–IV SCCHN, nimotuzumab significantly improved the complete response rate (59.5% vs. 34.2%; *p* = 0.028) and showed a delayed survival benefit (median OS: 12.5 vs. 9.5 months; *p* = 0.0491), which was more pronounced in EGFR-positive tumors (median OS: 16.5 vs. 7.2 months; *p* = 0.0038) [4].

The first post-registration trial explored nimotuzumab maintenance therapy beyond the six doses given concurrently with radiotherapy. Median survival was not reached in this patient subgroup, providing the rationale for an extended dosing regimen [5]. A subsequent phase IV study evaluated nimotuzumab in combination with radiotherapy, followed by maintenance [6]. In that study, the median survival in the nimotuzumab–radiotherapy group was 24.9 months, exceeding that reported in the controlled trial in which only six doses of nimotuzumab were administered [5,6,7]. While cross-trial comparisons are limited and not conclusive, longer exposure to nimotuzumab seemed to be associated with better survival.

In a phase II trial of 35 patients with recurrent/metastatic nasopharyngeal carcinoma after radical radiotherapy, first-line nimotuzumab (200 mg weekly) was added to cisplatin and 5-fluorouracil (every 3 weeks for up to six cycles). Median progression-free and overall survival were 7.0 and 16.3 months. Patients receiving ≥2400 mg of nimotuzumab had superior outcomes vs. those with lower exposure for objective response rate (88.9% vs. 12.5%, *p* < 0.001), median progression-free survival (7.4 vs. 2.7 months), and median overall survival (17.0 vs. 8.0 months) [8]. Likewise, in a different phase 2 trial in the same scenario, nimotuzumab was given with docetaxel and cisplatin every 3 weeks for six cycles (200 mg on days 1, 8, and 15 per cycle, totaling 18 doses). Patients receiving ≥2400 mg of nimotuzumab had significantly better progression-free survival (7.5 vs. 4.4 months) and overall survival (42.3 vs. 17.9 months) compared to those who received fewer nimotuzumab doses [9].

## 5. Esophageal Cancer

In a randomized trial of patients with inoperable locally advanced esophageal cancer, adding nimotuzumab to chemo-radiotherapy (cisplatin, fluorouracil, and external beam radiation) was safe and improved response rate and survival. Nimotuzumab was administered concurrently with chemoradiotherapy and then followed by weekly monotherapy for up to 26 cycles. The combined endoscopic and pathologic complete response rate was significantly higher with nimotuzumab (62.3% vs. 37.0%, *p* = 0.02), while the overall survival favored the nimotuzumab arm (median 15.9 vs. 11.5 months; HR 0.68), with no compromise in quality of life [10].

A previous controlled clinical trial evaluated only six doses of nimotuzumab in combination with the same scheme (cisplatin, fluorouracil, and external beam radiation) [11]. The complete response rate of the nimotuzumab arm was 26.1%, while the median survival time was 8.1 months [11]. As in the head and neck cancer setting, although comparing separate trials using the same regimen but different treatment durations is biased, longer-term nimotuzumab treatment appeared to improve tumor response and survival.

## 6. Pancreatic Indication

In patients with locally advanced or metastatic pancreatic cancer, the addition of nimotuzumab to gemcitabine improved PFS and survival vs. gemcitabine alone, with a favorable safety profile. The treatment scheme across three studies evaluated nimotuzumab at a fixed dose of 400 mg administered intravenously once weekly, combined with gemcitabine at 1000 mg/m^2^ on days 1, 8, and 15 of a 28-day cycle that continued until disease progression or unacceptable toxicity [12,13,14].

Data from phase IIb and III trials show that this combination significantly improves overall survival (OS) and progression-free survival (PFS), particularly in the KRAS wildtype subgroup. In the German phase IIb study, median overall survival was 8.6 vs. 6.0 months (HR = 0.69), with 1-year OS rates of 34% vs. 19% [14]. In a Cuban real-world study, the median OS was 9.0 months, rising to 16.4 months in locally advanced disease and 17.4 months in patients receiving eight or more nimotuzumab doses [13]. The pivotal Chinese phase III NOTABLE study, conducted exclusively in KRAS wildtype patients, confirmed a median OS of 10.9 vs. 8.5 months (RMST ratio 0.62, *p* = 0.036) [12].

The consistent survival benefit across diverse populations indicates that nimotuzumab plus gemcitabine is an active, well-tolerated first-line regimen for advanced pancreatic cancer, with the greatest benefit observed in KRAS wildtype patients. Unlike other anti-EGFR agents that have not shown efficacy in this disease, nimotuzumab’s favorable toxicity profile allows sustained treatment, a feature that may contribute to the survival benefit observed with extended administration. These findings indicate that nimotuzumab plus gemcitabine may represent a potential first-line treatment option for patients with KRAS wildtype advanced pancreatic cancer, although further validation in larger studies is needed [12,13,14].

## 7. High-Grade Glioma

In a randomized trial of high-grade glioma (41 anaplastic astrocytoma, 29 glioblastoma), nimotuzumab plus radiotherapy, given as six weekly induction doses followed by 1 year of maintenance every 3 weeks, significantly improved survival vs. radiotherapy and placebo (median 17.8 vs. 12.6 months) with an excellent safety profile [15]. In a separate randomized phase III trial of newly diagnosed glioblastoma, nimotuzumab added to radiotherapy and temozolomide, administered as 12 weekly doses of 400 mg during concurrent treatment, followed by 400 mg biweekly until progression, extended median overall survival. A larger benefit was observed in patients with MGMT-unmethylated tumors (19.5 vs. 15.5 months) [16].

## 8. Pediatric Glioma

In two separate studies conducted in children with central nervous system tumors in Cuba, nimotuzumab was administered as an induction phase of 150 mg/m^2^ weekly for 12 weeks, followed by maintenance doses every 14 days. This regimen was applied as monotherapy or combined with other treatments based on tumor type. Specifically, children with high-grade glioma (HGG) received nimotuzumab with radiotherapy and/or chemotherapy (prednisone, procarbazine, cyclophosphamide, and cisplatin), while those with progressive low-grade glioma (LGG) were treated with vincristine, carboplatin, and etoposide. For ependymomas, the combination included cyclophosphamide, vincristine, and etoposide, whereas children with brainstem gliomas (BSG) primarily received nimotuzumab with concomitant radiotherapy. The maximum treatment duration reached 4 years (48 months), with some patients receiving up to 108 doses of the antibody. In both studies, long-term nimotuzumab was exceptionally well tolerated, showing no cumulative toxicity or rebound effects after treatment cessation [17,18]. The most frequent adverse events were mild to moderate skin rash, mucositis, and vomiting; notably, no cardiac, hepatic, or renal impairments were detected, even in patients receiving more than 40 doses. Regarding efficacy, the therapy provided clinical benefits: in a trial conducted in 23 HGG patients, the 2-year survival rate was 54.2% with a median survival of 32.66 months, far exceeding historical controls. Across a broader cohort of 88 pediatric patients, nimotuzumab enabled prolonged disease stabilization and complete responses, with 16 children remaining progression-free 3 years after stopping treatment [17,18].

A phase II study evaluated the combination of nimotuzumab, vinorelbine, and radiotherapy in 25 children with newly diagnosed diffuse intrinsic pontine glioma (DIPG). Treatment consisted of nimotuzumab 150 mg/m^2^ and vinorelbine 20 mg/m^2^ administered weekly during the first 12 weeks with radiotherapy, followed by maintenance therapy with both agents every two weeks until disease progression or for 2 years. At week 12, the objective response rate was 96%. Median progression-free survival was 8.5 months, and median overall survival reached 15 months, with 1- and 2-year overall survival rates of 76% and 27%, respectively, comparing favorably with historical chemoradiotherapy regimens [19]. Consistent with these findings, a separate study in 40 pediatric patients with DIPG evaluating three first-line approaches—nimotuzumab–vinorelbine (including maintenance), temozolomide-based therapy (alone or combined with irinotecan and bevacizumab), and the VECC protocol (vincristine, etoposide, and carboplatin or cyclophosphamide—demonstrated that the nimotuzumab–vinorelbine regimen (including maintenance) was associated with the longest median overall survival (16 months), exceeding that observed with temozolomide-based regimens (10 months) and VECC (11 months) [14]. The nimotuzumab–vinorelbine combination including maintenance vs. vinorelbine will be further explored in children with relapsing diffuse and non-diffuse HGG, DIPG and gliomatosis cerebri.

## 9. Solid Tumors

In a retrospective analysis of 205 patients with advanced epithelial tumors treated in the real-world setting, long-term nimotuzumab treatment, defined as more than six weekly doses, was associated with improved PFS or OS across multiple indications, with a consistent safety profile characterized by minimal grade 3 or 4 toxicity and low rates of dermatologic adverse events [20]. In colorectal cancer (71 patients), receipt of more than six nimotuzumab doses was independently associated with prolonged progression-free survival (HR 0.40, *p* = 0.020), contributing to a median overall survival of 21.8 months [20]. In gastric cancer (35 patients), the use of more than six doses also correlated with improved overall survival in multivariate analysis (HR 0.15, *p* = 0.048 [20]. In esophageal cancer (21 patients), those treated with a higher number of doses had substantially better progression-free survival (1536 vs. 141 days; HR 0.19, *p* = 0.011) [20]. A randomized, double-blind trial in 118 patients with stage IVB, recurrent, or persistent cervical squamous cell carcinoma showed that adding nimotuzumab to paclitaxel–cisplatin followed by nimotuzumab maintenance every 2 weeks was significantly better, yielding a median overall survival of 15.7 vs. 12.4 months (HR = 0.72). The survival benefit was most pronounced in the recurrent disease scenario (OS = 21.7 vs. 12.4 months; HR = 0.62) [21]. These results support extending nimotuzumab therapy as maintenance beyond the initial combination treatment [21].

Collectively, these findings indicate that extended nimotuzumab exposure beyond six weekly doses confers a survival advantage across several advanced epithelial malignancies, warranting further prospective evaluation of maintenance strategies with this drug [20]. Table 2 summarizes the most important findings in relation to nimotuzumab maintenance.

Nimotuzumab binds to the extracellular domain of EGFR, specifically recognizing an epitope located within domain III that overlaps with both the cetuximab binding patch and the ligand-binding site [22].

## 10. Signal Transduction Inhibition

Both the murine parental antibody (ior-egf/r3) and its humanized derivative (nimotuzumab) inhibit EGFR signal transduction by blocking ligand-induced receptor autophosphorylation [23,24,25]. Evidence across multiple cell models consistently supports this mechanism. In H125 lung adenocarcinoma cells, ior-egf/r3 reduces EGFR autophosphorylation in a dose-dependent manner [23]. Similarly, in A431 cells, both ior-egf/r3 and nimotuzumab downregulate ligand-induced EGFR tyrosine phosphorylation [24]. Extending these findings, nimotuzumab also inhibits EGF-induced EGFR phosphorylation in a concentration-dependent manner in NSCLC cell lines with moderate or high EGFR expression (H292 and Ma-1), but not in low-expressing lines (H460, H1299, H1975). Taken together, these results indicate that the inhibitory effect of nimotuzumab on the EGFR signaling is closely associated with the level of receptor expression [24].

## 11. Consequences of the Signal Transduction Inhibition

### 11.1. Direct Antiproliferative Activity

In vitro assays using the EGFR-overexpressing A431 vulvar carcinoma cell line demonstrated that nimotuzumab exerts a potent antiproliferative effect [26]. Flow cytometric analysis of the cell cycle reveals that this antiproliferative effect is mediated by a G1 arrest accompanied by a corresponding decrease in the proportion of cells in S and G2 phases, consistent with the upregulation of cyclin-dependent kinase inhibitors such as p27Kip1, as described for other anti-EGFR antibodies [27]. Importantly, this direct effect in vitro is predominantly cytostatic rather than cytotoxic, as evidenced by the absence of a hypodiploid peak in DNA profiles [26].

### 11.2. Anti-Angiogenic Effect

EGFR signaling transcriptionally upregulates several pro-angiogenic factors, most notably vascular endothelial growth factor (VEGF) [26,28]. In A431 cells, treatment with nimotuzumab produces a dose-dependent downregulation of both VEGF mRNA and the secreted protein levels [26]. This effect is mechanistically specific, as nimotuzumab completely abrogates the increase in VEGF production induced by exogenous transforming growth factor-alpha (TGF-α) [26]. In xenograft models, subcutaneous A431 tumors treated with nimotuzumab exhibit a marked reduction in VEGF mRNA levels compared to control tumors. Immunohistochemical staining for the endothelial marker von Willebrand factor (vWF) revealed a striking reduction in microvascular density in treated tumors, along with a decrease in the caliber of remaining vessels and the disappearance of the large, complex “mother vessels” characteristic of VEGF-driven angiogenesis [26].

### 11.3. Pro-Apoptotic Effect

In standard monolayer cultures (in vitro), nimotuzumab fails to induce significant apoptosis [26]. Even in three-dimensional spheroid cultures, which more closely mimic the architecture of solid tumors, the maximum pro-apoptotic effect was a modest two-fold increase [26]. In sharp contrast, tumor xenografts harvested from mice treated with nimotuzumab show a dramatic five-fold increase in the apoptotic index, as measured by TUNEL staining, with apoptotic nuclei becoming abundant throughout the tumor parenchyma [26].

### 11.4. Effect on the Cancer Stem Cells

Nimotuzumab plays a role in the management of cancer stem cells (CSCs) by reducing their population density and enhancing their sensitivity to traditional therapies [29,30]. By blocking EGFR-mediated survival signals, nimotuzumab effectively depletes radioresistant CD133+ populations in glioma and acts as a potent radiosensitizer, overcoming the intrinsic resistance of CSCs to ionizing radiation [29,30]. Furthermore, the antibody disrupts vital intracellular pathways such as the PI3K/AKT axis, leading to the downregulation of stemness transcription factors, which are essential for CSC maintenance [29,30,31].

Beyond direct growth inhibition, nimotuzumab mitigates tumor progression by suppressing epithelial–mesenchymal transition (EMT) through the Akt/YB-1/AR signaling axis, thereby reducing the migratory potential associated with stem-like traits [31]. Consequently, the integration of nimotuzumab into clinical regimens offers a targeted strategy to address CSC-driven therapy resistance and improve long-term prognostic outcomes in malignancies such as glioblastoma and head and neck squamous cell carcinoma [30].

**Figure 1:**
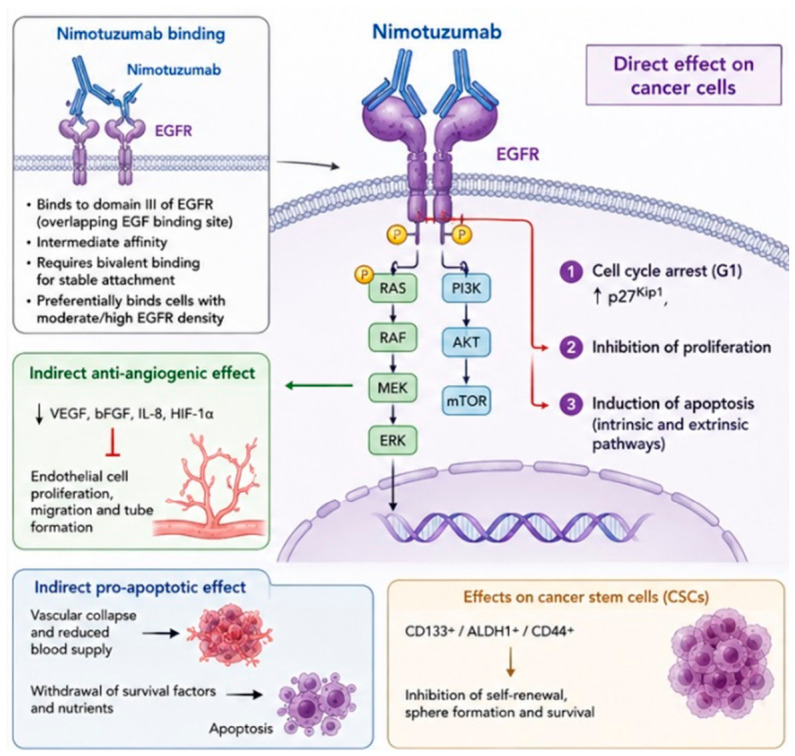
Integrated mechanism of action of nimotuzumab. This scheme depicts nimotuzumab’s multimodal antitumor effects: direct EGFR blockade inhibiting downstream proliferative and survival signaling (Ras/Raf/MAPK, PI3K/Akt); inhibition of angiogenesis; induction of apoptosis and effect on the cancer stem cells.

Figure 1 illustrates the mechanism of action of nimotuzumab described so far.

### 11.5. Effect on the Immune System

Beyond its direct anti-proliferative and anti-angiogenic effects, nimotuzumab exerts multiple immunomodulatory functions that contribute to its antitumor activity. As a humanized IgG1 monoclonal antibody, nimotuzumab engages innate immune effector cells by binding to Fcγ receptors [32,33]. It effectively induces antibody-dependent cellular cytotoxicity (ADCC), whereby natural killer (NK) cells lyse EGFR-positive tumor cells at levels comparable to cetuximab, despite nimotuzumab’s lower affinity for EGFR [33]. This ADCC activity is accompanied by CD16 downregulation on NK cells, confirming Fc receptor engagement [33]. Furthermore, nimotuzumab promotes NK cell activation, as evidenced by the upregulation of the activation markers CD69 and CD137 (4-1BB), as well as secretion of interferon-gamma (IFNγ) [33]. Importantly, nimotuzumab-activated NK cells facilitate dendritic cell (DC) maturation, increasing the expression of HLA-DR, CD83, and CD137L, and driving IL-12 secretion [33]. This NK-DC cross-talk leads to enhanced cross-priming of EGFR-specific CD8+ T cells, as demonstrated by increased frequencies of EGFR-tetramer-positive T cells in vitro and IFNγ-producing EGFR-specific T cells in long-term treated head and neck cancer patients in vivo [33]. Notably, while nimotuzumab upregulates the immune checkpoint molecules TIM-3 on NK cells and PD-L1 on DCs, this upregulation is significantly lower than that induced by cetuximab, suggesting a more favorable immunomodulatory profile with potentially reduced checkpoint-mediated suppression [33].

In addition to these effects on innate and adaptive immunity, nimotuzumab directly enhances tumor cell immunogenicity by upregulating human leukocyte antigen (HLA) class I expression [34]. Mechanistically, EGFR signaling suppresses HLA class I and antigen processing machinery (APM) components. Nimotuzumab treatment reverses this suppression, leading to a coordinated transcriptional increase in HLA class I heavy chains, beta-2-microglobulin (β2m), and multiple APM components including transporter associated with antigen processing (TAP), low molecular weight polypeptide (LMP), tapasin, calnexin, and endoplasmic reticulum (ER)p57, in both human and murine tumor cell lines [34]. In syngeneic mouse models, the surrogate anti-mouse EGFR antibody 7A7 increased HLA class I expression in tumors in vivo, which correlated with enhanced susceptibility to CD8+ T cell-mediated lysis and increased IFNγ production by tumor-specific T cells [34]. This effect was dependent on H-2Kb molecules, underscoring the role of HLA class I upregulation in improving tumor antigen presentation and T cell recognition. The magnitude of HLA class I upregulation by nimotuzumab is dependent on tumor EGFR expression levels, with higher EGFR density associated with stronger induction, consistent with the antibody’s density-selective binding properties [34].

Finally, nimotuzumab treatment influences regulatory T cell (Treg) dynamics in a context-dependent manner. In patients with locally advanced cervical cancer receiving concurrent chemo-radiotherapy (CCRT), the addition of nimotuzumab improved the balance of T lymphocyte subsets, increasing total T cells, while decreasing Tregs, compared to CCRT alone [35]. In head and neck cancer patients, nimotuzumab combined with cisplatin-based CCRT initially increased circulating CD4+CD39+Foxp3+ Tregs after the induction phase, likely due to the known resistance of Tregs to chemoradiation [33]. However, during subsequent nimotuzumab monotherapy maintenance (9–12 months), Treg frequencies decreased back to baseline levels [33]. This contrasts with cetuximab monotherapy, which has been reported to persistently increase Tregs. Apart from cetuximab, the frequency of circulating NK cells remained stable throughout nimotuzumab-based treatment [33,35]. Collectively, these findings indicate that nimotuzumab not only blocks EGFR signaling, but also orchestrates a broad immunostimulatory program, including antibody-dependent cell cytotoxicity (ADCC), NK-DC cross-talk, EGFR-specific T cell priming, and HLA class I upregulation, while exerting a neutral or favorable effect on regulatory T cell dynamics, thereby contributing to its durable clinical responses and low toxicity profile.

In addition, Hu and colleagues demonstrated that nimotuzumab inhibits the TGF-β-induced upregulation of the immune checkpoint CD276 at both transcriptional and protein levels, primarily by blocking the EGFR/MEK/ERK signaling pathway [36]. In xenograft and mouse head and neck squamous cell carcinoma (HNSCC) models, nimotuzumab suppressed tumor growth, reduced CD276 expression, and remodeled the tumor immune microenvironment by increasing the infiltration of cytotoxic T lymphocytes, helper T lymphocytes, and macrophages, while NK cells, dendritic cells, and neutrophils remained unchanged [36]. Previously, it has been demonstrated that CD276 is overexpressed in HNSCC tissues compared to normal tissue, and that high CD276 expression correlates with poorer patient survival and is negatively associated with tumor-infiltrating lymphocytes [36].

**Figure 2:**
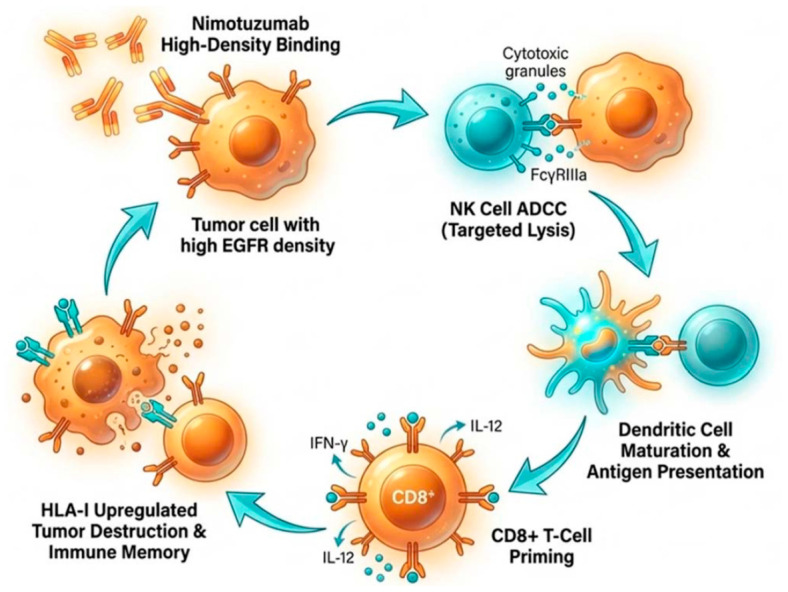
Nimotuzumab engagement of immune effector cells via Fcγ receptors to trigger antibody-dependent cellular cytotoxicity (ADCC) followed by activation of dendritic cells, leading to the induction of cytotoxic CD8 T-cells. In addition, nimotuzumab upregulates HLA-I expression in tumor cells.

Figure 2 describes the effect of nimotuzumab on the immune system.

## 12. Role of Nimotuzumab in Inflammation (The COVID-19 Experience)

Zhou et al. demonstrated that treatment with nimotuzumab reduced interleukin-6 (IL-6) levels in several pancreatic cancer cell models, both in vitro and in vivo [37]. In 2022, our group reported that EGFR was overexpressed in multiple lung cell populations from deceased patients with COVID-19, including type I and type II pneumocytes, alveolar macrophages and fibroblasts [38]. According Matsuyama et al. [39], SARS-CoV-2 induced lung injury together with STAT1 dysregulation, promoting EGFR overexpression [39]. This upregulation further increased the pro-inflammatory responses and prothrombotic state [39]. Treatment with nimotuzumab reduced the circulating levels of key inflammatory biomarkers, particularly interleukin 6 (IL-6) and the plasminogen activator inhibitor-1 (PAI-1), in both moderately and severely ill patients [40]. Overall, nimotuzumab was used to treat more than 1000 severe and critically ill COVID-19 patients. When compared with a propensity score-matched control group that received the same therapy without the antibody, nimotuzumab-treated patients had a twofold lower risk of death than those treated with steroids, anticoagulation and antibiotics [41]. The antibody also prevented lung fibrosis on follow-up imaging [38], suggesting that EGFR blockade can interrupt the interplay between hyperinflammation, coagulopathy and fibrosis in COVID-19.

## 13. Density Selective Targeting and Safety Profile

The most clinically relevant and distinguishing feature of nimotuzumab is its unique pharmacodynamic profile [42], which explains the striking absence of skin rash, a near-universal and often dose-limiting toxicity of other EGFR inhibitors such as cetuximab, panitumumab or other EGFR small tyrosine kinase inhibitors like erlotinib, afatinib, osimertinib and gefitinib [43]. This property is rooted in the antibody’s intermediate affinity for EGFR, which confers “density-selective targeting.”

According Garrido et al., nimotuzumab requires bivalent (two-arm) binding to achieve stable attachment to cells, whereas cetuximab binds strongly, even monovalently [44]. On high-EGFR-expressing tumor cells, both antibodies bind bivalently and accumulate to similar degrees, producing comparable antitumor effects in vitro and in vivo. However, on normal tissues such as skin and kidney, where EGFR density is low, cetuximab continues to bind strongly, while nimotuzumab’s monovalent interaction is transient and weak, resulting in minimal recognition of healthy cells [44]. Functionally, cetuximab reduces cell viability and induces apoptosis independently of EGFR expression, while nimotuzumab’s antitumor efficacy decreases as EGFR density declines. Thus, nimotuzumab’s intermediate affinity and dependence on bivalent binding for stable attachment explain its clinical profile: effective tumor targeting with low toxicity, refining the “affinity window” hypothesis [44].

A separate mathematical model consisting of four differential equations describing antibody distribution in plasma, tumor, liver, and skin compartments predicts that there exists an optimal affinity window for EGFR antagonists [45]. Specifically, antibodies with very high affinity (KD ≈ 10^−10^ M or lower) bind rapidly and stably to both tumor cells and normal epithelial cells (such as skin keratinocytes, which express low to moderate levels of EGFR), leading to receptor blockade in normal tissues and consequent toxicity [45]. Conversely, antibodies with very low affinity fail to achieve sufficient tumor binding. Antibodies with intermediate affinity (KD ≈ 10^−8^ to 10^−9^ M), like nimotuzumab, achieve maximum differential targeting: they accumulate on tumor cells that overexpress EGFR (where the high receptor density stabilizes binding) but interact only transiently with normal tissues expressing low receptor densities, resulting in rapid dissociation and minimal functional blockade [45].

**Figure 3:**
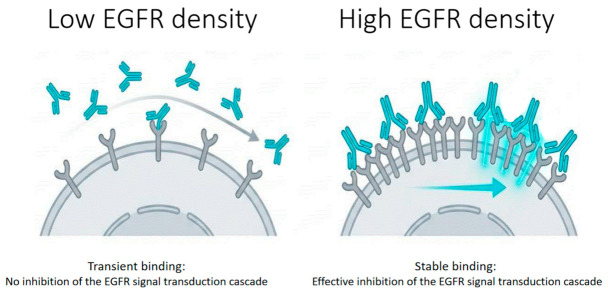
Nimotuzumab density selective targeting. Nimotuzumab binds transiently to cells with low epidermal growth factor receptor (EGFR) density, resulting in no inhibition of the downstream EGFR signal transduction cascade. In contrast, on cells with high EGFR density, nimotuzumab achieves stable binding, leading to effective blockade of the EGFR signaling cascade.

Figure 3 shows the differential EGFR binding dynamics of nimotuzumab as a function of receptor density.

This theoretical prediction has been validated in human pharmacodynamic trials. In a study of patients with unresectable squamous cell carcinoma of the head and neck who received a single infusion of nimotuzumab (200 or 400 mg) one week before starting radiotherapy, paired skin and tumor biopsies were analyzed for EGFR pathway activation [46]. Nimotuzumab reduced EGFR phosphorylation in basal keratinocytes but did not significantly affect downstream signaling, with *extracellular-regulated kinase* (ERK) and protein kinase B (AKT) activity remaining stable, together with a mild, nonsignificant decrease in proliferation [46]. Morphologically, skin biopsies showed no evidence of inflammatory changes: there was no vacuolar degeneration and no apoptotic keratinocytes [46]. In contrast, tumor samples exhibited reduced EGFR activation and decreased proliferation, along with a trend toward lower ERK activity. Notably, AKT phosphorylation increased in tumors after treatment, suggesting the activation of a potential feedback resistance mechanism and adaptive survival response [46].

Another clinical trial evaluating nimotuzumab biodistribution revealed consistent patterns across patients, with early accumulation in the liver, heart, kidneys, spleen, and bladder [3]. In contrast, tumor uptake and antibody retention expressed as the percentage of injected dose per gram of tissue were higher and more sustained in tumors compared to normal tissues, where activity declined after the first day [3]. These findings also confirmed selective and prolonged tumor targeting by nimotuzumab.

In summary, nimotuzumab works through multiple mechanisms, including direct G1 cell cycle arrest via EGFR blockade, potent anti-angiogenic effects mediated by VEGF downregulation, pro-apoptotic activity resulting from vascular collapse, and withdrawal of survival factors, specific effects of cancer stem cells and systemic immunomodulation through the stimulation of cytotoxic CD8^+^ T cells and downregulation of Tregs. Its intermediate affinity for EGFR enables density-selective targeting, achieving effective receptor blockade in EGFR-overexpressing tumors while sparing normal skin from the inflammatory consequences of sustained EGFR inhibition. This unique pharmacodynamic profile explains the absence of important toxicity in clinical trials and in the real-world scenario, involving over 500,000 patients.

## 14. Discussion

There is no single optimal duration that applies to all oncogene-addicted tumors. For metastatic tumors, the practical standard is continuous treatment, because the tumor often remains pathway-dependent and durable control is achieved while the target stays suppressed [47]. In particular, for tumors dependent on EGFR signaling, continuous receptor blockade is hypothesized to be necessary to maintain tumor growth inhibition and prevent repopulation. This rationale, taken from the use of other targeted monoclonal antibodies in oncology (e.g., trastuzumab, bevacizumab), supports administration until progression or even beyond [48,49]. For example, in HER2-positive metastatic breast cancer, optimal outcomes rely on continuous HER2 blockade across all lines of therapy. Standard first-line treatment combines trastuzumab, pertuzumab, and a taxane, followed at progression by newer HER2-targeted agents such as trastuzumab deruxtecan, and subsequently tyrosine kinase inhibitor-based or other HER2-directed regimens. Across the disease course, maintaining HER2 targeting, rather than switching to non-HER2 therapies, has consistently been associated with improved survival, supporting a treatment paradigm of sequential, sustained HER2 inhibition [50,51,52]. Likewise, the continuous or maintenance use of bevacizumab provides a sustained anti-angiogenic effect that translates into prolonged disease control across multiple tumor types. Remarkably, in metastatic colorectal cancer, long-term exposure, including continuation beyond progression, consistently improves progression-free and overall survival [53,54]. The standard practice is similar for small tyrosine kinase inhibitors targeting oncogenes. This concept is well established for EGFR-mutant and ALK-positive non-small-cell lung cancer, BCR-ABL chronic myeloid leukemia, and BRAF-mutant melanoma [55,56,57,58].

Still, another widely debated topic is the development of resistance after prolonged oncogene inhibition. In the context of EGFR blockade, preclinical research has uncovered several potential mechanisms underlying acquired resistance, including secondary mutations within the receptor’s extracellular domain (ECD) [59,60,61]; the compensatory activation of alternative receptor tyrosine kinases, particularly HER2, HER3 and MET [62,63]; and oncogenic mutations in the downstream pathway, including KRAS, NRAS, BRAF, and PIK3CA, that enable ligand-independent proliferation [64,65,66] and loss of PTEN tumor suppressor function [67].

In this context, nimotuzumab stands out as a unique anti-EGFR monoclonal antibody. Its antitumor effects arise from a complex mechanism combining direct antiproliferative activity, anti-angiogenic, pro-apoptotic, and immunomodulatory actions, along with a distinctive pharmacokinetic–pharmacodynamic profile that ensures a wide therapeutic index [3]. Nimotuzumab’s particular characteristics might lower the likelihood of resistance emergence. Thanks to its intermediate affinity and requirement for bivalent binding, the antibody preferentially targets cells displaying moderate to high EGFR expression, thus avoiding the intense selective pressure linked to complete pathway blockade. Collectively, these attributes suggest that nimotuzumab could reduce the evolutionary forces driving acquired resistance, positioning it as a valuable EGFR-targeting drug when long-term disease control is the primary goal. This hypothesis requires confirmation in properly designed preclinical and prospective clinical studies. Nimotuzumab also targets cancer stem cells, which are widely associated with resistance to chemotherapy and radiotherapy [68,69], and can induce a specific cytotoxic CD8 response, thereby acting as an active immunotherapy rather than solely as a targeted therapy. To elicit a sustained CD8 response while maintaining regulatory T cell control, extended treatment with nimotuzumab is required.

EGFR overexpression is not only a hallmark of epithelial tumors, but is also present in non-malignant cells within the tumor microenvironment (TME), where it promotes tumor growth [70]. For instance, endothelial cells express EGFR, which works together with VEGFR signaling to drive angiogenesis [71]. EGFR is essential for tumor-associated macrophage (TAM) tumor recruitment and polarization into an immunosuppressive M2-like phenotype [72]. Regulatory T cells (Tregs) express EGFR, and its activation enhances their suppressive function [73]. Myeloid-derived suppressor cells (MDSCs) rely on EGFR signaling to maintain their immature state and produce reactive oxygen species (ROS), dampening immunity [74]. In cancer-associated fibroblasts (CAFs), EGFR overexpression, driven by TGF-beta, promotes tumor invasion [75]. Apart from cancer, normal cells expressing EGFR in the TME are genetically stable and therefore unlikely to evade the selective pressure exerted by the antibody. Recent single-cell transcriptomic and spatial profiling studies support a broader role for EGFR-targeted therapies in reshaping the TME through multiple effects in the stromal, immune and vascular compartments [74,76,77,78]. Taniguchi et al. showed that colorectal tumors responding to anti-EGFR antibodies differ in cancer cells and also in the surrounding microenvironment [78]. Tumor-derived epiregulin activates EGFR-high fibroblasts, which drive myeloid cells toward an M2 state, linking EGFR blockade response to coordinated effects across the tumor, fibroblasts, and macrophages [78]. Zhao et al. similarly found that, in pancreatic adeno-squamous carcinoma, EGFR signaling extends beyond tumor cells to fibroblasts and myeloid cells, highlighting EGFR as a very relevant therapeutic target that also modifies the tumor microenvironment [77]. Distinguishing the direct pharmacologic effects of EGFR inhibition on the tumor microenvironment from indirect effects mediated through tumor cells remains an area of ongoing investigation. Our preliminary data from COVID-19 patients (a non-cancer scenario) suggest that nimotuzumab can have direct effects on cells overexpressing EGFR, including pneumocytes, fibroblasts and alveolar macrophages. In patients with severe and critical COVID-19, nimotuzumab reduced inflammatory markers, lowered mortality and prevented the development of pulmonary fibrosis [38]. Whether nimotuzumab can directly reshape the tumor stroma remains to be rigorously established.

Across heterogeneous studies, prolonged treatment with nimotuzumab was consistently associated with favorable outcomes. Most available evidence derives from retrospective studies that are susceptible to selection bias, confounding and immortal time bias. Consequently, the apparent benefits of prolonged treatment should be interpreted as exploratory and non-conclusive. Prospective studies specifically evaluating optimal duration and maintenance schedules are needed before generalized recommendations can be made.

EGFR-targeting therapies including small-molecule tyrosine kinase inhibitors (TKIs) and EGFR-recognizing antibodies act primarily by disrupting receptor signaling and the downstream proliferative, pro-survival, and pro-angiogenic pathways that sustain tumor growth. Antibodies like cetuximab, panitumumab, and nimotuzumab bind the extracellular domain of EGFR and prevent ligand-induced activation, whereas first-, second-, and third-generation TKIs (gefitinib, erlotinib, afatinib, osimertinib) inhibit the intracellular kinase domain.

Cetuximab and panitumumab are high affinity antibodies that bind EGFR on both tumor cells and normal cells, producing the characteristic acneiform rash in most patients (80–90%, with 10–16% grade 3–4) and hypomagnesemia (14–55%, with 6–17% grade 3–4) due to EGFR blockade in renal tubules [79,80]. Nimotuzumab, by contrast, has intermediate affinity and requires bivalent engagement, which restricts binding to cells with high EGFR density [44]. This selective uptake spares normal skin and kidney, resulting in markedly lower dermatologic toxicity and minimal electrolyte disorders [81]. The three antibodies also differ in isotype. Only the IgG1 molecules, nimotuzumab and cetuximab, activate Fcγ-receptor immune pathways [82]. Nimotuzumab induces strong NK-cell mediated ADCC, CD16 downregulation, IFNγ release, dendritic cell maturation, and priming of EGFR-specific CD8^+^ T cells, with only modest induction of inhibitory molecules such as TIM-3 and PD-L1 [33]. Cetuximab also elicits Fc-dependent immune activation, driving NK and neutrophil-mediated ADCC, high IFNγ secretion, DC maturation, enhanced antigen-processing machinery (TAP-1/2), and efficient cross-presentation of EGFR and other tumor antigens, ultimately expanding EGFR-specific CTLs in patients [83]. Panitumumab, an IgG2 antibody, effectively blocks EGFR signaling but shows minimal NK activation, ADCC or DC maturation, and does not expand EGFR-specific T cells in patients [82]. Unlike antibodies, TKIs do not engage Fc receptors and therefore do not activate innate or adaptive immune effector mechanisms. TKIs also generate higher rates of resistance, as they preferentially target mutated intracellular EGFR and lack immune mediated cytotoxicity [84]. According our premise, prolonged treatment appears to be the best strategy for EGFR targeting. The greatest benefit would come from antibodies with immunomodulatory capacity and affinities that favor tumor-selective binding over normal tissue recognition. Intermediate affinity, IgG1 subclass antibodies like nimotuzumab may have the best therapeutic index when considering long-term disease control, safety, and reduced resistance induction.

For nimotuzumab, future research priorities include prospective randomized trials specifically evaluating maintenance strategies, biomarker-driven patient selection, the molecular characterization of resistance mechanisms, integration with immunotherapy and modern combination regimens, and the development of next-generation products. The binding affinity of nimotuzumab was recently fine-tuned by our group using phage display [85]. The resulting monoclonal antibodies (K4 and K5) have higher affinity than nimotuzumab while remaining within the theoretically optimal intermediate pharmacodynamic range, still below cetuximab and panitumumab [85]. These improved nimotuzumab variants are currently under preclinical evaluation and have not yet advanced to clinical studies [86]. Furthermore, multiple antibody–drug conjugates (ADCs) incorporating nimotuzumab with various linkers and cytotoxic payloads are being investigated in preclinical settings [87,88]. Nimotuzumab has also been conjugated to several radioisotopes for applications in radioimmunotherapy and theragnostics [89,90,91,92,93]. Additionally, a chimeric antigen receptor T-cell (CAR-T) construct was developed using the nimotuzumab Fab fragment [94]. This CAR-T was compared with a cetuximab-based CAR-T in glioma xenograft mouse models. Both constructs demonstrated antitumor activity in tumors with high EGFR expression. Notably, the nimotuzumab-based CAR-T showed reduced activity in tumors with low EGFR expression, suggesting a potentially improved safety profile by minimizing on-target, off-tumor toxicity. As noted by the authors, “the weakness of the nimotuzumab-CAR affinity, is its apparent strength” [94].

Regarding new tumor indications, further investigation is warranted in locally advanced squamous cell carcinomas where concurrent chemoradiotherapy remains the standard first-line treatment. In this context, nimotuzumab could be administered concomitantly with chemotherapy and radiotherapy, followed by a consolidation maintenance phase. Beyond its currently approved use in head and neck and esophageal cancers, the therapeutic potential of nimotuzumab could be explored in other locally advanced squamous malignancies, including non-small-cell lung cancer (squamous subtype), anal cancer and cervical cancer. This strategy is supported by the high EGFR overexpression across squamous tumors and the favorable safety profile of nimotuzumab, which is unlikely to compromise treatment adherence. When combined with chemoradiotherapy, nimotuzumab may function as both a chemosensitizer and a radiosensitizer, potentially enhancing the efficacy of definitive local treatment. Continued administration as maintenance therapy could sustain EGFR blockade, thereby improving the control of residual disease and reducing the risk of recurrence.

In addition, considering the potential “vaccinal effect” of the antibody, an attractive clinical strategy would be to combine nimotuzumab with immune checkpoint inhibitors. This approach may be particularly relevant in the metastatic or recurrent setting of squamous carcinomas, where anti-PD-1 or anti-PD-L1 therapies are currently recommended, either as monotherapy or in combination with chemotherapy. Potential indications include squamous tumors of the head and neck, esophagus, skin, and non-small-cell lung cancer (squamous subtype).

## 15. Conclusions

In summary, the available evidence, although derived primarily from retrospective studies and protocols incorporating maintenance treatment, suggests the advantages of the prolonged use of nimotuzumab in the advanced cancer setting. The argument for long-term use is strongly supported by nimotuzumab’s favorable safety profile, particularly the very low incidence of severe skin toxicity and hypomagnesemia. However, the apparent benefits of prolonged treatment should not be interpreted as practice-changing, but rather as hypothesis-generating. Future optimization will likely replace fixed treatment durations with personalized strategies guided by measurable residual disease, circulating tumor DNA dynamics, and the characterization of resistance biomarkers. Besides the prospective, comparative clinical trials intended to show the superiority of maintenance treatment, optimization strategies also include improved formulations (e.g., subcutaneous administration), personalized combination regimens, and the development of next-generation approaches such as nimotuzumab-based antibody–drug conjugates, and multi-specific antibodies.

## Figures and Tables

**Table 1:** Nimotuzumab registered indications in Cuba.

Indication	Treatment Scheme
Locally advanced squamous cell carcinoma of the head and neck	Combination with radiotherapy or chemo/radiotherapy
Locally advanced nasopharyngeal tumors	Combination with radiotherapy or chemo/radiotherapy
Adult high-grade glioma (glioblastoma and anaplastic astrocytoma)	Combination with radiotherapy
Pediatric high-grade glioma (newly diagnosed)	Combination with radiotherapy and chemo-radiotherapy
Recurrent/refractory pediatric glioma	As monotherapy or in combination with chemotherapy
Locally advanced esophageal cancer	Combination with chemo-radiotherapy
Locally advanced/metastatic pancreatic adenocarcinoma	Combination with chemotherapy
Stage IV non-small-cell lung cancer, after front line therapy	Monotherapy, as switch maintenance
Treatment of patients with moderate and severe COVID-19 pneumonia	Combination with standard therapy according the patient condition

**Table 2:** Clinical evidence supporting prolonged nimotuzumab therapy in different tumor types. Across multiple malignancies, prolonged nimotuzumab administration was consistently associated with improved clinical outcomes, particularly overall survival, compared with shorter treatment schedules or standard therapy. OS: overall survival; CRR: complete response rate.

Indication	Treatment Regimen	Key Findings on Long-Term Treatment Benefit
Squamous cell carcinoma of the head and neck (locally advanced, unfit for concurrent radio-chemotherapy)	Trial 1: Radiotherapy + 6 weekly nimotuzumab doses Trial 2: Radiotherapy + 6 weekly nimotuzumab followed by maintenance until PD	Nimotuzumab maintenance showed longer survival than 6 doses OS: 24.9 vs. 12.5 months
Esophageal cancer (locally advanced)	Trial 1: Chemoradiotherapy + nimotuzumab (6 doses, induction) Trial 2: Chemoradiotherapy + nimotuzumab induction followed by weekly maintenance up to 26 cycles	Nimotuzumab maintenance showed longer survival and CRR than 6 doses OS: 15.9 vs. 8.1 months CRR: 62.3% vs. 26%
Metastatic pancreatic cancer (all patients or KRAS wildtype)	Trial 1 (all patients): Gemcitabine + weekly nimotuzumab until progression Trial 1 (KRAS wildtype): Gemcitabine + weekly nimotuzumab until progression	Maintenance was associated with longer survival in 2 controlled clinical trials. Trial 1(all patients): OS: 8.6 vs. 6.0 months Trial 2 (KRAS wildtype): OS: 10.9 vs. 8.5 months
Adult high-grade glioma (newly diagnosed)	Trial 1: Radiotherapy + 6 weekly nimotuzumab doses + maintenance every 3 weeks for 1 year. Trial 2: Radiotherapy + temozolomide + 12 weekly doses + biweekly maintenance until progression	Nimotuzumab maintenance was associated with longer OS in 2 controlled clinical trials. Trial 1 (HGG): OS 17.8 vs. 12.6 months Trial 2 (GBM, MGMT-unmethylated): OS 19.5 vs. 15.5 months
Pediatric glioma (newly diagnosed/relapsed)	Trial 1: Radio-chemotherapy + 12 weekly nimotuzumab + maintenance every 2 weeks Trail 2: Nimotuzumab/vinorelbine in relapsed DIPG	Nimotuzumab maintenance was associated with prolonged OS in 2 single-arm clinical trials: Trial 1 (newly DIPG): OS: 15 months Trial 2 (relapsed DIPG): OS: 16 months

## Data Availability

No new data were created or analyzed in this study. Data sharing is not applicable to this article.
